# Personal Resources for Psychological Well-Being in University Students: The Roles of Psychological Capital and Coping Strategies

**DOI:** 10.3390/ejihpe14100177

**Published:** 2024-10-02

**Authors:** Esteban Moreno-Montero, María del Mar Ferradás, Carlos Freire

**Affiliations:** Department of Psychology, University of A Coruña, 15071 A Coruña, Spain; e.morenom@udc.es (E.M.-M.); mar.ferradasc@udc.es (M.d.M.F.)

**Keywords:** psychological well-being, psychological capital, coping strategies, personal resources, university students

## Abstract

In recent years, research has noted the increasing prevalence of mental health problems among university students. The current mental health needs of the university population, along with the multitude of stressors they face, have increased the importance of examining their psychological well-being and determining the personal resources that effectively promote it. In this context, the present research aims to analyze the roles of psychological capital (PsyCap) and coping strategies as personal resources that are significantly related to the psychological well-being (PWB) of university students. Specifically, the mediating roles of various coping strategies (both adaptive and maladaptive) in the relationship between PsyCap and PWB are explored. The study involves 391 university students from Spain. The results show partial mediation effects of adaptive coping strategies (cognitive restructuring and social support) on the relationship between PsyCap and PWB. Likewise, PsyCap is shown to be a direct positive predictor of adaptive coping strategies and PWB, as well as a direct negative predictor of maladaptive coping strategies (self-criticism). Therefore, it is concluded that PsyCap and some adaptive coping strategies serve as valuable personal resources that predict PWB in university students. PsyCap is also associated with a lower tendency to engage in maladaptive coping strategies, such as self-criticism. Similarly, the use of cognitive restructuring and/or social support is related to high levels of PWB among university students.

## 1. Introduction

The scientific study of psychological well-being has gained considerable interest in recent decades. In this context, university students are one of the populations that are attracting the most attention due to the association of this educational stage with possible risks to mental health. Several meta-analyses highlight the increasing prevalence of depression and anxiety in university students [1,2], especially after the COVID-19 pandemic [3,4]. Facing academic demands, being challenged by their economic status, adapting to a new context, establishing new social networks, and obtaining employment all contribute to a complex web of stressors [5,6]. Students are forced to deal with these challenges simultaneously, which negatively affects their academic engagement and performance and increases university dropout rates [7,8].

The current mental health needs of university students, as well as the multitude of stressors they face, demonstrate the importance of examining psychological well-being in this population and determining effective psychological resources for its promotion. To this end, the present study analyzes the relationships among psychological capital, the coping strategies employed by students in response to the various stressful situations they face, and psychological well-being. Specifically, the aim is to determine whether psychological capital favors the use of adaptive coping strategies that enhance psychological well-being.

### 1.1. Psychological Well-Being: The Focus on Human Flourishing

The concept of well-being is multifaceted, with varying interpretations across diverse contexts. Two distinct perspectives are currently the most relevant in psychological research [9]. Subjective well-being, which is based on hedonistic philosophy, has the components of the search for pleasurable experiences that grant satisfaction and the predominance of positive affectsover negative ones [10]. The eudaimonic perspective focuses on psychological well-being and is based on personal development and optimal and purposeful functioning in an environment [11].

Since the emergence of positive psychology, the study of psychological well-being has become particularly relevant. From this neophyte perspective, human flourishing is emphasized as the most genuine exponent of mental health [12], which is understood as a desired vital state that integrates hedonic and eudaimonic aspects of well-being [13]. Although various models of psychological well-being representing human flourishing have been proposed, they all converge in the identification of dimensions such as meaning and purpose in life, positive relationships with others, engagement, environmental mastery, self-acceptance, and positive affect [14]. Thus, this conceptualization of well-being puts a focus on positive psychological functioning, the absence of pathology, and the pursuit of pleasurable experiences [15,16].

Given that it represents the essence of psychological well-being, in recent years, there has been unprecedented research on human flourishing. Regarding university students, studies have yielded interesting findings on the correlations of psychological well-being with other adaptive academic variables. For example, Datu [17] and Datu et al. [18] identified the direct positive impacts of psychological well-being on perceived performance, learning goal orientation, and delayed gratification. In the same vein, Howell [19] evidenced that students with higher levels of psychological well-being showed greater tendencies toward self-regulated learning. In addition, psychological well-being has been associated with high levels of self-compassion and purpose in life [20] and with improvements in the mental health and emotional awareness [21] of university students in different cultural contexts.

### 1.2. Coping Strategies

Lazarus and Folkman’s transactional theory of stress and coping [22] and the Job Demands–Resources (JD-R) model [23] provide a solid conceptual framework when explaining the underlying mechanism of well-being among students. Although the JD-R model was initially proposed in the work context, its applicability has been evidenced in students [24]. According to this model, the well-being experienced by a student is the result of the balance between contextual demands and the environmental and personal resources available to cope with the demands. The relationship between demands and resources triggers the development of two opposing psychological processes. If the demands (e.g., work overload and time pressure) exceed the resources to cope with them, a health impairment process begins. As a result, the student gradually begins to feel “burned out” in their academic performance, which has a negative impact on their physical and psychological health. On the contrary, if the resources are effective in responding to the demands, the motivational process is triggered, which leads to the experience of high levels of engagement; this, in turn, favors psychological well-being.

Although the relevance of environmental resources (e.g., social support, feedback, and autonomy) is not minor, the JD-R model emphasizes the significance of personal resources [25]. Within them, as suggested by the transactional theory of stress and coping, the strategies employed by the student to cope with the demands have a preponderant role. Coping strategies refer to cognitive, emotional, and/or behavioral efforts to address (master, reduce, or tolerate) a troubled person–environment relationship [26]. Accordingly, coping strategies play a crucial long-term role in students’ psychological well-being [27].

In response to the innumerable strategies used by humans to cope with stressful events, different taxonomies have been proposed. One of the most widespread is the one that classifies coping strategies into two major typologies [28]: approach and avoidance. The former encompasses those cognitive and behavioral efforts aimed at providing an active response to the stressor, directly modifying the problem (primary control) or the negative emotions derived from it (secondary control). This group includes planning, the adoption of a specific action, support-seeking (instrumental and emotional), positive re-evaluation, and acceptance. Avoidance coping strategies refer to the cognitive and/or behavioral mechanisms used to avoid stressful situations, such as distraction, denial, or desiderative thinking.

In the academic context, approach-type coping strategies are related to good academic, physical, and psychological adjustments [29,30]. Thus, adaptive coping strategies show positive and significant correlations with the eudaimonic components of well-being, such as self-acceptance and purpose in life [31,32]. In addition, adaptive coping strategies favor engagement and academic performance in university students [33]. In contrast, avoidant coping strategies often involve maladaptive consequences for students [34,35], such as higher levels of interpersonal stress [36] and burnout in university students [37].

In summary, the research shows a clear relationship between coping strategies and the level of psychological well-being experienced by university students [38]. However, it is necessary to determine the positive personal resources that have an effect in promoting adaptive coping strategies over maladaptive ones. It is also worth analyzing whether the relationship between personal resources and coping strategies affects the psychological well-being of university students.

### 1.3. Personal Resources: Psychological Capital

Psychological Capital (PsyCap) was proposed as a paradigmatic exponent of positive organizational behavior [39]. PsyCap is comprised of four psychological resources—self-efficacy, hope, optimism, and resilience—which act synergistically to influence psychological well-being [40]. Indeed, a recent meta-analysis [41] has shown that PsyCap correlates positively with desirable attitudes in the work environment, such as satisfaction and engagement. Moreover, PsyCap constitutes a robust predictor of job performance, given that it influences workers’ adaptability, competence, and proactivity. In contrast, PsyCap is negatively related to cynicism, stress, and anxiety [42].

Research agrees that PsyCap is also highly functional in the academic context. For example, some studies have shown that PsyCap is a decisive mediator in the relationships between positive emotions [43], academic engagement [44], and the teacher–student relationship [45] with academic performance. It has also been evidenced that PsyCap directly influences performance [46] and academic adjustment in the university stage [47]. Moreover, PsyCap has emerged as a highly adaptive resource in terms of psychological well-being [48,49]. In addition, PsyCap is shown to be a predictor of adaptive coping in university students [50].

### 1.4. The Present Study

Research considers PsyCap as a possible personal resource with great potential for psychological well-being. However, to date, there is little existing research on the subject concerning university students. At this academic stage, students deal with numerous daily demands that may threaten their psychological well-being, requiring the use of adaptive coping strategies. Based on these claims, it can be assumed that students with high PsyCap develop more adaptive coping strategies in the face of daily demands, and both aspects (PsyCap and adaptive coping strategies) contribute jointly to psychological well-being. This postulate is consistent with the Conservation of Resources (COR) theory [51], according to which people who possess personal resources (e.g., PsyCap) show high motivation to acquire, maintain, and promote new resources in coping with demands (adaptive coping strategies); this spiral of positive gains will yield long-term adaptive personal outcomes (e.g., higher psychological well-being).

**Figure 1:**
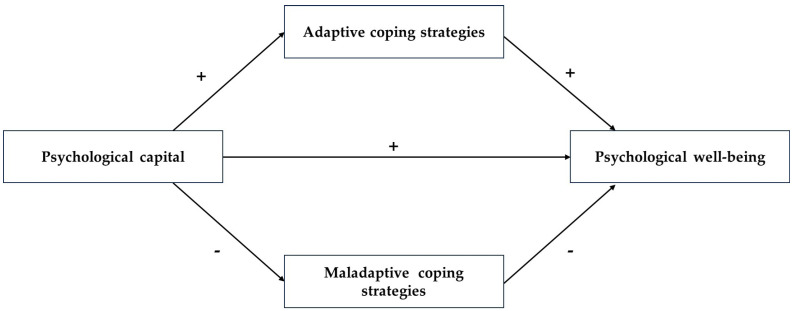
Hypothesized relationships between PsyCap, coping strategies, and psychological well-being.

Based on the reviewed studies, the present research aims to identify whether PsyCap and coping strategies function as personal resources for psychological well-being in university students. As specific objectives, we propose to (a) examine the relationship between PsyCap and psychological well-being, (b) determine the relationship between PsyCap and coping strategies, (c) estimate the specific role played by individual coping strategies (both adaptive and maladaptive) in their relationship with psychological well-being, and (d) analyze the mediating role of coping strategies in the relationship between PsyCap and psychological well-being. Based on the preceding research, the following hypotheses are put forward (see Figure 1):

**H1.**  *PsyCap is a direct positive predictor of psychological well-being*.

**H2.**  *PsyCap is a direct positive predictor of adaptive coping strategies, as well as a direct negative predictor of maladaptive coping strategies*.

**H3.**  *Adaptive coping strategies positively predict psychological well-being*.

**H4.**  *Maladaptive coping strategies negatively predict psychological well-being*.

**H5.**  *Coping strategies partially mediate the relationship between PsyCap and psychological well-being*.

## 2. Materials and Methods

### 2.1. Procedure

The research was conducted in accordance with the principles and standards established in the Declaration of Helsinki and the Code of Ethics of the University of A Coruña. The study was approved by the Academic Committee of the Master’s Degree in Applied Psychology Program of the University of A Coruña (MUPA/2024/01/29). The data collection process was carried out between February and April 2024. The instruments were distributed through an online survey generated via Microsoft Forms. The link was shared through the students’ institutional e-mail. To prevent common method bias, various Likert response scales were employed, and participant anonymity was ensured. Therefore, the questionnaire included an informed consent section that provided students with details about the research and asked for voluntary participation, with a guarantee of anonymity and confidentiality in their responses. Additionally, it was emphasized that there were no right or wrong answers to prevent socially desirable responses. The average time to complete the survey was 13 min.

### 2.2. Participants

A non-probabilistic convenience sampling was used to select participants, with the following inclusion criteria: (a) voluntary participation under explicit informed consent, (b) enrollment as an undergraduate or master’s student, and (c) being over 18 years of age. The number of participants was initially 438 students belonging to the University of A Coruña (Spain). After excluding random and incongruent participations (specifically those with a response time of less than 3 min or more than 40 min), the final sample consisted of 391 university students. The ages of male (24.30%; *M* = 24.44, *SD* = 6.68), female (71.61%; *M* = 23.46, *SD* = 6.25), and other-gender (4.09%; *M* = 21.50, *SD* = 3.14) participants ranged from 18 to 59 years old.

The majority were undergraduate students belonging to the Social and Legal Sciences program (*n* = 132, 33.76%), followed by Master’s (*n* = 103, 26.34%), Engineering and Architecture (*n* = 67, 17.13%), Health Sciences (*n* = 40, 10.23%), Experimental Sciences (*n* = 28, 7.16%), and Arts and Humanities (*n* = 21, 5.37%) program students. Most of the participants were in their final year (*n* = 106, 27.11%), followed by first-year (*n* = 89, 22.76%), second-year (*n* = 49, 12.53%), and third-year (*n* = 46, 11.77%) students. In addition, most of the participants did not combine their studies with a job (*n* = 276, 70.59%), studied full-time (*n* = 348, 89%), did not receive a scholarship (*n* = 217, 55.50%), and had middle (*n* = 192, 49.11%) and lower-middle (*n* = 125, 31.97%) socioeconomic status. Other socio-demographic and academic characteristics of the sample are presented in Table 1.

### 2.3. Instruments

The Psychological Capital in Academic Contexts Questionnaire (PCQ-12) was used to measure PsyCap. PCQ-12 is the adaptation of the original PCQ by Luthans et al. [52], and it was validated in the Spanish academic context by Martínez et al. [53]. It is a self-administered instrument that aims to measure the four components of the PsyCap: self-efficacy (3 items), hope (4 items), resilience (3 items), and optimism (2 items). An example statement is, “I feel confident sharing information about my studies with other people”. For its scoring, a seven-option Likert scale was used, where participants rated their level of agreement or disagreement between 0 (“Completely disagree”) and 6 (“Completely agree”). For interpretation, the scores of each item were summed, such that the higher the score was, the higher the level of academic PsyCap. The factorial validity of the instrument was evaluated by taking into consideration the following parameters [54]: the fulfillment of non-significance (*p* > 0.05) in the case of the Chi-square (χ^2^); the Chi-square to the degrees of freedom (df) ratio less than 3; the attainment of values greater than 0.95 for the Goodness of Fit Index (GFI), the Adjusted Goodness of Fit Index (AGFI), the Comparative Fit Index (CFI), and the Tucker–Lewis Index (TLI); and the attainment of values less than 0.08 in the Root Mean Square Error of Approximation (RMSEA) and 0.06 in the Standardized Root Mean Residual (SRMR). In addition, the internal consistency of the instrument was determined using the McDonald’s omega (ω) statistic. In the present study, the questionnaire showed excellent psychometric properties, both in factorial validity (χ^2^ = 140.16; df = 49; *p* < 0.05; GFI = 0.98; AGFI = 0.96; TLI = 0.93; CFI = 0.95; SRMR = 0.054; and RMSEA = 0.07) and internal consistency (ω = 0.92; 95% CI [0.90, 0.93]).

Coping strategies were assessed using the Coping Strategies Inventory (CSI). It is the modified version adapted to Spanish by Cano et al. [55] from the Coping Strategies Inventory [56]. The instrument included 40 items that measure the use of eight coping strategies (five items per strategy, e.g., “I went over the problem again and again in my mind, and in the end, I saw things in a different way”). Four of the strategies were adaptive: problem-solving (PSO), emotional expression (EEX), social support (SSU), and cognitive restructuring (CRE). The other four were maladaptive strategies: self-criticism (SCR), desiderative thinking (DTH), problem avoidance (PAV), and social withdrawal (SWI). The instrument was self-administered using a Likert scale of five options from 0 (“Not at all”) to 4 (“Totally”). The participants indicated the degree to which they used each strategy. The scores of the five items of each factor were added together, giving a minimum score of 0 and a maximum of 20. A higher score indicated a higher degree of the strategy under consideration. In the present study, both the factor structure of the model representing Adaptive Coping Strategies (PSO, EEX, SSU, and CRE) and that of the model representing Maladaptive Coping Strategies (SCR, DTH, PAV, and SWI) evidenced acceptable fits: χ^2^ = 512.391; df = 166; *p* < 0.05; GFI = 0.94; AGFI = 0.92; TLI = 0.89; CFI = 0.90; SRMR = 0.08; RMSEA = 0.07; and χ^2^ = 512.391; df = 166; *p* < 0.05; GFI = 0.93; AGFI = 0.90; TLI = 0.83; CFI = 0.85; SRMR = 0.09; and RMSEA = 0.08, respectively. Regarding internal consistency, adaptive coping strategies showed excellent values (ω = 0.90; 95% CI [0.86, 0.91]), and maladaptive coping strategies achieved good scores (ω = 0.86; 95% CI [0.84, 0.88]). Values for individual coping strategies were as follows: PSO (ω = 0.85; 95% CI [0.82, 0.87]), SCR (ω = 0.88; 95% CI [0.86, 0.90]), EEX (ω = 0.87; 95% CI [0.85, 0.89]), DTH (ω = 0.84; 95% CI [0.81, 0.87]), SSU (ω = 0.88; 95% CI [0.86, 0.90]), CRE (ω = 0.83; 95% CI [0.80, 0.86]), PAV (ω = 0.73; 95% CI [0.69, 0.77]), and SWI (ω = 0.82; 95% CI [0.79, 0.84]).

Psychological well-being was measured by means of the Flourishing Scale. The instrument was developed by Diener et al. [57] and adapted to the Spanish context by Checa et al. [58]. It consisted of eight items that measured psychological well-being in various areas, such as social relationships or daily activities (e.g., “I see my future with optimism”). The scale was self-administered and scored on a seven-option Likert scale, with a range between 1 (“Strongly disagree”) and 7 (“Strongly agree”). It was scored by the sum of the answers in the eight items, which made it possible to obtain a total of 56. The higher the score was, the higher the level of well-being. In the present study, the psychometric properties of the scale were adequate in terms of factorial validity (χ^2^ = 65.97; df = 20; *p* < 0.05; GFI = 0.99; AGFI = 0.99; TLI = 0.92; CFI = 0.94; SRMR = 0.042; and RMSEA = 0.08) and internal consistency (ω = 0.86; 95% CI [0.84, 0.88]).

### 2.4. Data Analysis

The statistical analysis consisted of two sections. In the first section, the descriptive statistics of the study variables were calculated. Measures such as the arithmetic mean (*M*) and standard deviation (*SD*) were obtained. In addition, compliance with normality was assumed when the values of dispersion, skewness, and kurtosis were in the range between [−1.5, +1.5] [59]. For the analysis of coping strategies, the results were obtained for each individual strategy and for the groups of adaptive (PSO, EEX, SSU, and CRE) and maladaptive (SCR, DTH, PAV, and SWI) coping strategies. In this section, we also obtained the Pearson correlation matrix between PsyCap, coping strategies, and psychological well-being. To identify the specific role of each coping strategy, a multiple regression analysis was conducted (stepwise method). Age, gender, and PsyCap were also included in the regression analysis. In this way, predictors that were not significant for the model were eliminated in the mediation analysis.

In the second section, a multiple mediation analysis was performed by constructing a Structural Equation Model (SEM). The Maximum Likelihood (ML) estimator and the Bootstrapped Confidence Intervals method of 5000 cases with a 95% confidence interval were used to ensure the robustness and precision of our parameter estimates [60]. In the SEM, the direct and indirect effects of PsyCap on psychological well-being were estimated through adaptive coping strategies and maladaptive coping strategies. All analyses were performed using the R programming language version 4.3.1 [61] and the packages ‘psych’ [62], ‘Lavaan’ [63], and ‘semhelpinghands’ [64].

To confirm the goodness of fit of the SEM model, the following parameters were taken into consideration [54]: the fulfillment of non-significance (*p* > 0.05) in the case of the Chi-square; the attainment of values less than 0.08 in the RMSEA and 0.06 in the SRMR; and the attainment of values greater than 0.95 for CFI and TLI.

## 3. Results

### 3.1. Descriptive Analyses

Table 2 shows the descriptive results of the variables. Apart from psychological well-being, all variables conformed to the assumption of normality in terms of skewness and kurtosis. The trends of PsyCap and psychological well-being toward the upper end of the range of possible responses suggested a moderate-to-high presence of these factors in the sample. Regarding coping strategies, the data indicate a pronounced tendency to use DTH (*M* = 13.88; *SD* = 4.67), followed by PSO (*M* = 12.31; *SD* = 4.17). In contrast, PAV (*M* = 7.37; *SD* = 4.44) and EEX (*M* = 9.68; *SD* = 4.96) were the least used coping strategies. In addition, the averages of adaptive coping strategies and maladaptive coping strategies were relatively similar, with a slight predominance of adaptive coping strategies (*M* = 43.86; *SD* = 13.89).

### 3.2. Correlational Analyses

Table 3 shows the results of the correlations between the variables assessed in the sample. The correlations between PsyCap and adaptive coping strategies were positive and significant (*p* < 0.001) and ranged from *r*_(PsyCap–EEX)_ = 0.22 to *r*_(PsyCap–PSO)_ = 0.42. In addition, the association between PsyCap and the total construct of adaptive coping strategies was positive and moderate (*r*_(PsyCap–Adaptive Coping)_ = 0.43, *p* < 0.001). On the other hand, the correlations between PsyCap and maladaptive coping strategies were inverse and significant (*p* < 0.001), lying in the range between *r*_(PsyCap–DTH)_ = −0.19 and *r*_(PsyCap–SCR)_ = −0.34. The only non-significant relationship was with PAV (*r* = −0.05, *p* = 0.30). As for the correlation between PsyCap and the total construct of maladaptive coping strategies, it was moderate and negative (*r*_(PsyCap–Maladaptive coping)_ = −0.30, *p* < 0.001). The correlation between PsyCap and psychological well-being (PWB) was significant, high, and positive (*r*_(PsyCap–PWB)_ = 0.65, *p* < 0.001).

Regarding the relationships between psychological well-being and coping strategies, all were significant (*p* < 0.001), except for the correlation with PAV (*r* = −0.04, *p* = 0.46). Correlations with individual adaptive coping strategies ranged from *r*_(PWB–EEX)_ = 0.23 to *r*_(PWB–PSO and SSU)_ = 0.43. In the case of individual maladaptive strategies, the correlations were within the range *r*(_PWB–DTH)_ = −0.16 and *r*_(PWB–SCR)_ = −0.30. Accordingly, the association between psychological well-being and the total adaptive coping strategies factor was positive and moderate (*r*_(PWB–Adaptive coping)_ = 0.51, *p* < 0.001). However, the correlation with the total factor of maladaptive coping strategies was inverse and low (*r*_(PWB–Maladaptive coping)_ = −0.27, *p* < 0.001).

### 3.3. Multiple Regression Analysis

The results of the multiple regression analysis are shown in Table 4. The adaptive coping strategies that predicted psychological well-being were cognitive restructuring (CRE) and social support (SSU). In contrast, among the maladaptive coping strategies, only self-criticism (SCR) was shown to be a significant predictor of psychological well-being. Regarding the roles of PsyCap, age, and gender, the latter was excluded from the model since it did not achieve statistical significance. The model including these variables (CRE, SSU, SCR, PsyCap, and age) explained 50% of the overall variance, and its fit was adequate (R^2^ = 0.50, F = 79.8, and *p* < 0.001).

### 3.4. Multiple Mediation Analysis

As indicated in Table 5, PsyCap was shown to be a positive predictor of psychological well-being (*b* = 0.655, 95% CI [0.593, 0.711]). When analyzing indirect relationships, the results indicated that the two adaptive coping strategies (cognitive restructuring and social support) played significant mediating roles in the relationship between PsyCap and psychological well-being (*b* = 0.117, 95% CI [0.077, 0.159]). In contrast, the indirect relationship through self-criticism (maladaptive coping strategy) did not show statistical significance (*b* = 0.028, 95% CI [−0.001, 0.059]). PsyCap was a positive and significant direct predictor of adaptive coping strategies (*b* = 0.378, 95% CI [0.275, 0.468]). Conversely, PsyCap showed a negative and significant relationship with self-criticism (*b* = −0.334, 95% CI [−0.429, −0.236]).

Moreover, adaptive coping strategies directly and positively predicted psychological well-being (*b* = 0.309, 95% CI [0.240, 0.377]), whereas self-criticism showed a significant negative direct relationship with psychological well-being (*b* = −0.082, 95% CI [−0.162, 0.003]). Finally, the findings suggested that the mediating effect of coping strategies was partial because PsyCap showed a direct positive relationship with psychological well-being, even after accounting for indirect pathways (*b* = 0.511, 95% CI [0.431, 0.587]). In addition, the variable age only had a weak and negative relationship with psychological well-being (*b* = −0.080; 95% CI [−0.156; −0.010]). However, the relationship with coping strategies was not significant.

**Figure 2:**
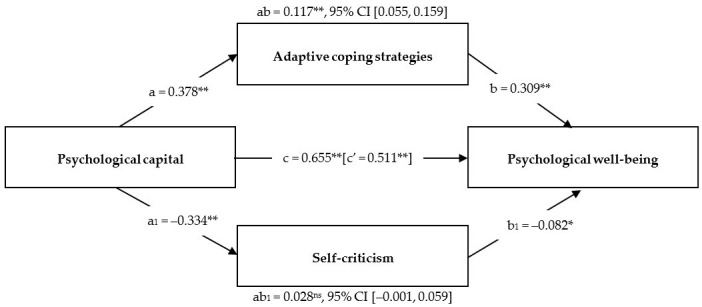
Graphical representation of multiple mediation analysis. Note. * *p* < 0.05; ** *p* < 0.001; and ns = non-significant.

The proposed SEM model is shown graphically in Figure 2. The fit indices indicated that the model was adequate (χ^2^(1) = 1.002; *p* > 0.05; RMSEA = 0.002; SRMR = 0.012; CFI = 0.99; and TLI = 0.99). As in Table 5, it was observed that the mediation of coping strategies was partial.

## 4. Discussion

The aim of the present research was to analyze the relationships among PsyCap, coping strategies, and psychological well-being in university students. Specifically, it was explored whether the relationship between PsyCap and psychological well-being was partially mediated by coping strategies.

Consistent with the first hypothesis, the results evidenced that PsyCap was a direct and positive predictor of psychological well-being. In other words, the availability of high standards of hope, optimism, self-efficacy, and resilience in university students was directly linked to experiencing everyday life in terms of flourishing. These findings validated the theoretical basis of PsyCap, which was shown to be a positive predictor of psychological well-being [39]. They were consistent with studies highlighting the positive association between PsyCap and subjective and psychological well-being among students [48,49,65].

The results also confirmed the second hypothesis, whereby it was expected that PsyCap would be a direct positive predictor of adaptive coping strategies and a direct negative predictor of maladaptive coping strategies. This finding seemed to indicate that the higher the PsyCap of a university student was, the greater their tendency to cope with diverse and heterogeneous daily demands by using adaptive strategies. At the same time, a high PsyCap level was related to a decrease in the use of maladaptive strategies in the face of daily stressors, particularly self-criticism. Thus, these results aligned with the scarce previous existing evidence regarding the positive relationship between PsyCap and adaptive coping strategies in university students [46,50]. They also endorsed the inverse relationship between PsyCap and maladaptive coping strategies, as evidenced in previous work [66,67,68]. Based on these findings, PsyCap appeared to stand as an important personal resource in the face of the daily demands of university students, given that it was not only associated with the use of adaptive strategies but also linked to a decrease in the use of dysfunctional strategies.

As a third hypothesis, the present study postulated that adaptive coping strategies would be a direct positive predictor of psychological well-being. The results obtained ratified this expectation so that, in line with the findings of other studies [31,32], the valuable role played by adaptive coping with stress on students’ psychological well-being seemed to be confirmed. In this regard, one of the main contributions of the present study lay in the identification of individual adaptive coping strategies that were significant predictors of psychological well-being. Our findings indicated that cognitive restructuring and social support strategies were significantly related to psychological well-being at the university stage. In other words, experiencing high levels of psychological well-being was related to students’ ability to value the positive side of problems and to seek emotional and instrumental support in difficult situations.

However, our fourth hypothesis was only minimally endorsed. Specifically, we only found a significant relationship between one of the assessed maladaptive coping strategies (self-criticism) and psychological well-being. This finding suggested that frequent self-criticism would be related to a significant decrease in psychological well-being. However, the use of other dysfunctional coping strategies to deal with stressful situations did not predict (neither positive nor negative) psychological well-being. Although studies such as Rabenu et al. [66] already found the absence of a significant relationship between avoidance strategies and workers’ psychological well-being, this finding could have a possible explanation. In this way, it was necessary to consider that well-being was not equivalent to the absence of ill-being [69]. Therefore, it could not be ruled out that the frequent use of maladaptive coping strategies was related to the experience of distress in the long term, as indicated by some studies [70].

Finally, the fifth hypothesis was only partially confirmed since we found a mediation effect of adaptive coping strategies (but not of maladaptive strategies) on the relationship between PsyCap and psychological well-being. According to this result, high levels of PsyCap were related to the greater use of adaptive coping strategies (specifically, cognitive restructuring and social support), which allowed one to effectively manage and overcome stressful circumstances and, consequently, to experience high psychological well-being. To the best of our knowledge, this finding was not confirmed in students, although it was consistent with other research involving occupational samples [66,68]. Our results also aligned with those of Wang et al. [71], who established that PsyCap and positive reappraisal (an adaptive coping strategy) mediated the relationship between family support and psychological well-being.

The research conducted had implications of theoretical and applied relevance. From the theoretical point of view, it seemed to endorse the proposition that positive personal resources (in this case, the PsyCap components—optimism, hope, resilience, and self-efficacy—and adaptive coping strategies) functioned as mechanisms that improved the psychological well-being of university students. This finding would be consistent with the Conservation of Resources (COR) Theory, which suggests that people tend to acquire and conserve personal resources, as their accumulation decreases the impact of stress [51]. In this context, the resources forming the PsyCap not only promoted the adoption of adaptive coping strategies to deal with stressful situations but also appeared to function as a reservoir of resources protecting psychological well-being. From a practical standpoint, the findings of this study suggested that interventions focused on PsyCap components and adaptive coping strategies could have positive effects on the psychological well-being of university students. These types of interventions have already evidenced their effectiveness in both work [39] and academic [72] contexts.

Regarding the latter, various methods could be employed to improve university students’ psychological capital and cultivate positive psychological functioning. Zeng et al. [73] advocated that higher education institutions should (a) promote students’ development of flexible life goals (i.e., setting challenging objectives that encourage students to strive for and achieve their aspirations); (b) foster students’ achievement orientation in their areas of interest; (c) cultivate innovative thinking (this way of thinking would stimulate curiosity, propels students to actively seek knowledge, and contributes to fortifying the tenacious volitional skills in the face of obstacles); and (d) cultivate positive self-reflective awareness (self-reflection would prompt students to objectively assess self-questions and accomplishments through introspection. Thus, students could develop an optimistic outlook on life). In addition, students’ psychological capital could also be enhanced by encouraging them to express their opinions via teamwork and by guiding them to deal with difficulties and pressure in an adaptive way [74].

In turn, an adequate response to daily demands could also be favored by workshops specifically designed for learning adaptive coping strategies. In recent years, interventions aimed at improving the coping skills of university students have proliferated. Most of these initiatives have adopted both cognitive-behavioral and mindfulness-based approaches [75,76,77]. In these programs, students would learn to identify the signs of stress, as well as the external (environmental demands) and internal (thoughts, emotions) factors participating in its development. Furthermore, students would acquire a wide range of adaptive coping strategies (e.g., planning, problem-solving, positive reappraisal, and meditation) and metacognitive abilities to evaluate and select the best coping strategies in each situation [78,79].

Without underestimating its relevance, the results of the present study should be considered in light of its limitations. First, the sample was not selected by a probabilistic method and was concentrated only on students from one Spanish university. This limitation made it difficult to generalize the findings to the entire population of university students. Therefore, future research should replicate the results of the present study using more rigorous sampling procedures in other cultural contexts. Second, the cross-sectional nature of the study did not allow for the establishment of causal relationships between variables. Thus, longitudinal research analyzing the long-term influence of PsyCap and coping strategies on the psychological well-being of university students would be needed. Finally, the use of self-reported instruments could introduce biases in the results (e.g., social desirability, overestimation, or underestimation of behavior).

## 5. Conclusions

The psychological well-being of university students has become a major focus in recent years. In accordance with this salutogenic approach [80], the main contribution of the present study is the identification of PsyCap (self-efficacy, optimism, hope, and resilience) and adaptive coping strategies (social support and cognitive restructuring) as factors that are significantly related to higher levels of psychological well-being in university students. Specifically, our results show that PsyCap is directly and indirectly (via social support and cognitive restructuring) associated with psychological well-being. Likewise, PsyCap is negatively related to the use of a deniably dysfunctional coping strategy, such as massive self-criticism. These findings suggest that psychological well-being and adaptive coping with everyday demands are associated with a high PsyCap. More importantly, the use of adaptive coping strategies (particularly cognitive restructuring and social support) is a valuable psychological resource that, in conjunction with PsyCap, contributes to high levels of psychological well-being at the university stage.

## Figures and Tables

**Table 1:** Sociodemographic and academic characterization of the sample.

Variables	*n* = 391	% Sample
**Gender**		
Male	95	24.30
Female	280	71.61
Other	16	4.09
**Age**		
Range	18–59	
Mean	23.62	
**Career Field**		
Arts and Humanities	21	5.37
Engineering and Architecture	67	17.13
Experimental Sciences	28	7.16
Health Sciences	40	10.23
Master	103	26.34
Social and Legal Sciences	132	33.76
**Study Year**		
Year 1	87	22.25
Year 2	49	12.53
Year 3	46	11.77
Year 4	106	27.11
Master	103	26.34
**Grades**		
1–4	10	2.55
5	21	5.37
6	86	22
7–8	220	56.27
9–10	54	13.81
**Socioeconomic Level**		
Low	20	5.12
Lower-middle	125	31.97
Middle	192	49.10
Upper-middle	51	13.04
High	3	0.77
**Work and Study**		
Yes	115	29.41
No	276	70.59
**Full-time Student**		
Yes	348	89
No	43	11
**Scholarships**		
Yes	174	44.50
No	217	55.50

Note. Range of grades: 1–4 = failing grade; 5–6 = bare pass; 7–8 = outstanding; and 9–10 = excellent.

**Table 2:** Descriptive statistics of the variables.

Variables	*M*	*SD*	Skewness	Kurtosis	ω
Psychological Capital	47.51	15.17	−0.70	0.06	0.92
Problem-Solving	12.31	4.17	−0.17	−0.35	0.85
Self-Criticism	11.31	5.21	−0.01	−0.94	0.88
Emotional Expression	9.68	4.96	0.17	−0.80	0.87
Desiderative Thinking	13.88	4.67	−0.55	−0.49	0.84
Social Support	11.42	5.09	−0.08	−0.93	0.88
Cognitive Restructuring	10.45	4.55	0.14	−0.74	0.83
Problem Avoidance	7.37	4.44	0.40	−0.38	0.73
Social Withdrawal	9.69	5.08	0.20	−0.87	0.82
Adaptive Coping Strategies	43.86	13.89	0.05	−0.27	0.90
Maladaptive Coping Strategies	42.25	13.87	0.04	−0.34	0.86
Psychological Well-Being	44.04	7.28	−1.14	1.84	0.86

Note. Range of possible PsyCap scores (0–72); Range of possible PSO to SWI scores (0–20); Range of possible adaptive and maladaptive coping strategies scores (0–80); Range of possible psychological well-being scores (8–56); *M* = mean; and *SD* = standard deviation.

**Table 3:** Correlation matrix of the variables.

Variables	1	2	3	4	5	6	7	8	9	10	11	12
1 PsyCap	-											
2 PSO	0.42 **	-										
3 SCR	−0.34 **	−0.13 *	-									
4 EEX	0.22 **	0.29 **	0.00 ^ns^	-								
5 DTH	−0.19 **	−0.08 ^ns^	0.51 **	0.21 **	-							
6 SSU	0.30 **	0.35 **	−0.14 *	0.53 **	0.03 ^ns^	-						
7 CRE	0.36 **	0.51 **	−0.17 **	0.24 **	−0.12 *	0.44 **	-					
8 PAV	−0.05 ^ns^	0.00 ^ns^	0.14 *	−0.16 **	0.18 **	−0.08 ^ns^	0.25 ^**^	-				
9 SWI	−0.25 **	−0.14 *	0.43 **	−0.33 **	0.32 **	−0.43 **	−0.13 *	0.47 **	-			
10 ACS	0.43 **	0.70 **	−0.14 **	0.72 **	0.02 ^ns^	0.81 **	0.73 **	0.00 ^ns^	−0.36 **	-		
11 MCS	−0.30 **	−0.13 **	0.75 **	−0.10 *	0.70 **	−0.22 **	−0.07 ^ns^	0.60 **	0.79 **	−0.18 **	-	
12 PWB	0.65 **	0.43 **	−0.30 **	0.23 **	−0.16 **	0.43 **	0.44 **	−0.04 ^ns^	−0.25 **	0.51 **	−0.27 **	-

Note. PSO = Problem-Solving; SCR = Self-Criticism; EEX = Emotional Expression; DTH = Desiderative Thinking; SSU = Social Support; CRE = Cognitive Restructuring; PAV = Problem Avoidance; SWI = Social Withdrawal; ACS = Adaptive Coping Strategies; MCS = Maladaptive Coping Strategies; PWB = Psychological Well-Being; ns = no significance; * *p* < 0.05; and ** *p* < 0.001.

**Table 4:** Multiple regression analysis, taking Psychological Well-Being as the dependent variable.

Variables	B	SE B	β	R^2^	F
**Step 1**				0.41	278 **
PsyCap	0.31	0.02	0.65 **		
**Step 2**				0.46	168 **
PsyCap	0.27	0.02	0.56 **		
CRE	0.37	0.06	0.23 **		
**Step 3**				0.49	127 **
PsyCap	0.25	0.02	0.53 **		
CRE	0.25	0.07	0.16 **		
SSU	0.29	0.06	0.21 **		
**Step 4**				0.50	97.5 **
PsyCap	0.24	0.02	0.50 **		
CRE	0.24	0.07	0.15 **		
SSU	0.29	0.06	0.20 **		
SCR	−0.11	0.05	0.08 *		
**Step 5**				0.50	79.8 **
PsyCap	0.25		0.51 **		
CRE	0.24		0.15 **		
SSU	0.30		0.21 **		
SCR	−0.12		−0.08 *		
Age	−0.09		−0.08 *		

Note. PsyCap = Psychological Capital; CRE = Cognitive Restructuring; SSU = Social Support; SCR = Self-Criticism; * *p* < 0.05; and ** *p* < 0.001.

**Table 5:** Standardized multiple mediation analysis results.

Variables	Coeff.	SE	z	P	Lower CI	Upper CI
**Direct Effect**						
PsyCap → PWB	0.511	0.040	12.859	0.001	0.431	0.587
Age → PWB	−0.080	0.037	−2.139	0.032	−0.156	−0.010
PsyCap → ACS	0.378	0.049	7.783	0.001	0.275	0.468
Age → ACS	0.065	0.046	1.394	0.163	−0.024	0.156
PsyCap → SCR	−0.334	0.050	−6.743	0.001	−0.429	−0.236
Age → SCR	−0.043	0.044	−0.986	0.324	−0.128	0.045
ACS → PWB	0.309	0.034	8.969	0.001	0.240	0.377
SCR → PWB	−0.082	0.041	−2.031	0.042	−0.162	−0.003
**Indirect Effect**						
PsyCap → ACS → PWB	0.117	0.021	5.533	0.001	0.077	0.159
PsyCap → SCR → PWB	0.028	0.015	1.862	0.063	−0.001	0.059
**Total Effect**	0.655	0.030	21.608	0.000	0.593	0.711

Note. PsyCap = Psychological Capital; PWB = Psychological Well-Being; ACS = Adaptive Coping Strategies (Cognitive Restructuring and Social Support); SCR = Self-Criticism; and CI = Confidence Interval (95%).

## Data Availability

The study data are available in the Zenodo repository (https://doi.org/10.5281/zenodo.12774887). The dataset can be accessed upon request to the contact author of the manuscript. The data presented in this study are available on request from the corresponding author.
